# Vulnerability to stress, anxiety and depressive symptoms and metabolic control in Type 2 diabetes

**DOI:** 10.1186/1756-0500-5-271

**Published:** 2012-06-07

**Authors:** Carlos Gois, Vasco V Dias, João F Raposo, Isabel do Carmo, Antonio Barbosa

**Affiliations:** 1 Department of Psychiatry, Faculty of Medicine, University of Lisbon, Santa Maria Hospital, Av. Professor Egas Moniz, Lisbon, 1600, Portugal; 2 Portuguese Diabetes Association (APDP); Medical School, New University, Lisbon, Portugal; 3 Department of Endocrinology, Diabetes and Metabolism, Faculty of Medicine, University of Lisbon, Santa Maria Hospital, Lisbon, Portugal

**Keywords:** Type 2 diabetes mellitus, Vulnerability to stress, Metabolic control, Depressive symptoms, Anxiety symptoms

## Abstract

**Background:**

Vulnerability to stress has been associated to distress, emotional distress symptoms and metabolic control in type 2 diabetes mellitus (T2DM) patients as well. Furthermore some conflicting results were noticed. We aimed to evaluate the effect over metabolic control in what concerns vulnerability to stress beyond depressive and anxiety symptoms.

**Findings:**

This cross-sectional study assessed 273 T2DM patients with depressive and anxiety symptoms using the Hospital Anxiety Depression Scale (HADS) and the 23 Questions to assess Vulnerability to Stress (23QVS), along with demographic and clinical diabetes-related variables. Hierarchical logistic regression models were used to investigate predictors of poor glycemic control. The results showed an association of depressive symptoms (odds ratio = 1.12, 95%CI = 1.01-1.24, P = 0.030) with increased risk of poor glycemic control. Anxiety symptoms and vulnerability to stress on their own were not predictive of metabolic control, respectively (odds ratio = 0.92, 95%CI = 0.84-1.00, P = 0.187 and odds ratio = 0.98, 95%CI = 0.95-1.01, P = 0.282).

**Conclusions:**

Our data suggested that vulnerability to stress was not predictive of poor glycemic control in T2DM, but depressive symptoms were.

## Findings

### Background

Concept of stress concerns to an acute or chronic stressful event itself or either the biological or psychological response to such conditions [1]. In a major chronic disease such as type 2 Diabetes Mellitus (T2DM), stress comes potentially from two cumulative sources, namely, specific diabetes-related psychological distress, associated with repetitive intrusive treatment regimens or disabling chronic complications, and the generic daily life psychological stress of living with a chronic disease in a complex world with professional and interpersonal challenges [2].

Vulnerability to stress represents a general tendency to experience intense episodes of distress and negative affects and it is also a risk factor for depression or anxiety disorders [3-5]. Patients with T2DM with more vulnerability to stress, such as anxiety, neuroticism, timidity, less resilience, less social support and lower standards of living have been associated to contradictory results concerning the glycemic control [6-14]. Anxiety and depression have also been related to metabolic control [15-18] although some recent studies have yielded conflicting results [19-21]. Depressive symptoms and diabetes-specific distress are correlated to each other, although only specific distress displayed more linkages with behavioral and glycemic control [21-27]. In this sense, it seems that affective symptoms covariate with the negative emotional component of diabetes-specific distress, although one cannot always notice disturbances in what concerns metabolic control [19].

The aim of this study was to evaluate whether vulnerability to stress has any additional value beyond depressive or anxiety symptoms on glycemic control.

### Methods

#### Sample

This study was included in a screening protocol to detect depressive and anxiety states in patients with T2DM who have been followed up at the Portuguese Diabetes Association (APDP).

Diagnostics for patients with T2DM had to be established for at least 6 months earlier and followed up at the APDP for at least another 3 months. Patients’ age ranged from 18 to 65, and all had to be able to fill in the questionnaires and previously tested for HbA1c control. Patients with history of cerebrovascular disease were excluded. While waiting for their regular diabetes consultation 317 consecutive patients with T2DM were invited to participate in this study after detailed explanation.

The study was approved by local Ethics Committee of the APDP and all participants provided prior written informed consent in accordance with the ethical standards of the Helsinki Declaration.

#### Measures

A clinical interview was carried out with all patients, and information was collected concerning demographic and clinical data, namely body mass index (BMI), HbA1c level, diabetes therapy and antidepressant medication. All patients filled in clinical questionnaires. Chronic complications due to diabetes were identified by taking each patient’s medical history, symptoms, physical-evaluation findings and automated data into account. Medical records were used to check and complete data.

##### The hospital anxiety depression scale (HADS)

This self-report scale was designed to detect depressive and anxiety symptoms, and has 14 questions, seven on anxiety and seven on depression having only an answer to each question along a 0 to 3 points scale [28]. Final score ranges from 0 to 21 points for each sub-scale and a higher score means the presence of increased anxious or depression symptoms. A score ranging 0 to 7 is “normal”; from 8 to 10, “light”; from 11 to 14 “moderate” and from 15 to 21 “serious”. The version used was a Portuguese edition with Cronbach’s alpha = 0.81 for the depression sub-scale and 0.76 for anxiety. A sub-group of 316 patients with T2DM was included in the validation sample [29]. HADS continuous scores were used in our analysis.

##### The 23 questions to assess vulnerability to stress (23QVS)

This self-rating scale assesses the vulnerability to generic stress [30]. Through a literature review, 64 questions related to stress vulnerability facets were obtained. These facets were: negative and positive personality characteristics, regular exercise, confident availability, social and family support and adverse life conditions. Items were suppressed to obtain the final version based on: (a) absence of significant positive correlation with the neuroticism factor in Portuguese version of the Eysenck Personality Inventory (Eysenck, 1964) and psychopathology index at the Portuguese version of Brief Symptom Inventory (Derogatis, 1982) and absence of significant negative correlation with positive coping score from Problems Resolution Inventory, a Portuguese coping questionnaire (Vaz Serra, 1988) [31]; (b) significant differences by gender; (c) homogeneity problems, detected by Cronbach α changes after removing an item; (d) ceiling effects of some questions. Final version comprises 23 questions requiring only an answer for each question out of a range of 0 to 4; some items earns direct scores, while others scored reversely. Highest score always indicates greatest vulnerability to stress. Reliability and validity were performed on a sample of 368 subjects from general population. Cronbach’s alpha coefficient was 0.82 and test-retest r = 0.816, P < 0.001. Using factor analysis, seven main factors came out explaining 57.5% variance: Factor 1 – Perfectionism and intolerance to frustration, e.g. “I feel bad when I don’t excel in what I do”; Factor 2 – Inhibition and functional dependence, e.g. “I’m more likely to complain about routine set-backs than make an effort to solve them”; Factor 3 – Lack of social support, e.g. “When I’ve got a problem I’ve usually got someone to help me”(reverse score); Factor 4 – Adverse living conditions, e.g. “I have enough money to take care of my personal needs”(reverse score); Factor 5 – Dramatization of existence, e.g. “I’m the sort of easy-going person who makes a joke out of my mishaps”(reverse score); Factor 6 – Subjugation, e.g. “I spend more time helping others than I spend on my own needs”; Factor 7 – Deprivation of affection and rejection, e.g. “I’ve got unpleasant traits that put others off”. The 23QVS includes both individual and social vulnerability factors, which is considered to be a faster, more practical way of detecting vulnerability to stress instead of applying several scales to each factor. The scale comprising scores was fed into a computerized program devised by the test’s author. The 23QVS continuous score was used in our analysis.

The HbA1c values were determined at APDP using ion-exchange high-performance liquid chromatography, in a BIO-RAD Variant II turbo kit (according to the manufacturing data range 4.9–6.2%). HbA1c values were divided into 2 groups (<8% and ≥8%) to describe glycemic control for each subject, in accordance to previous reports [24,32,33].

#### Statistical analysis

All variables were described by undertaking the whole sample and the comparison between subgroups of glycemic control (HbA1c <8% and ≥ 8%). Student’s *t*-test and chi-square test were used to compare demographic, clinical and psychometric variables to all the patients in univariate analyses when the sample was divided to dichotomized HbA1c.

To evaluate the risk of poor glycemic control associated to vulnerability to stress beyond depressive or anxiety symptoms, a logistic hierarchical regression analysis was used. Analyses were performed with HbA1c dichotomized as dependent variable (≥8% = 1) and variables were entered in several blocks according to demographic variables, clinical variables, depressive and anxiety symptoms and vulnerability to stress. To check for multicollinearity we performed Pearson correlations on independent variables prior to logistic regressions analyses.

Odds ratio were used instead of size effects because we were dealing with logistic regression analysis [34]. A *P* value <0.05 was used to determine the level of statistical relevance. Statistical analysis was carried out using the SPSS 15.0 (SPSS Inc., Chicago, Illinois).

### Results and discussion

#### Patients’ characteristics

The study sample comprised a group of two-hundred and seventy three T2DM patients (139 men and 134 women). A total of 317 patients were invited and 28 were not eligible: short follow up at APDP (n = 9), recent T2DM diagnosis (n = 2), unable to fill in questionnaires (n = 4), previous stroke (n = 1), missing HbA1c level (n = 12). From eligible patients, 7 refused to participate (response rate = 97.5%). Their demographic features (age, gender and education) were not different from those included in the study. Due to missing data, 9 patients were further eliminated from analysis.

Participant patients with worse metabolic control were younger (mean = 59.15, SD = 4.69 vs. mean = 56.74, SD = 6.26, t = 3.63, P < 0.001), insulin users (43.0% vs. 68.1%, *X*^2^ = 16.82, P < 0.001) with higher score on HADS depression (mean = 3.93, SD = 3.11 vs. mean = 4.7, SD = 3.84, t = −2.00, P = 0.047). (Table 1)

**Table 1:** Sample description

	**Total sample**	**Patients by glycemic control level**		
	**N = 273**	**N = 107**	**N = 166**	
		**HbA1c < 8**	**HbA1c ≥ 8**	**P**
Age (years)	57.68 ± 5.81	59.15 ± 4.69	56.74 ± 6.26	**0.001**
Gender (male)	139 (50.9)	54 (50.5)	85 (51.2)	1.000
Education level (years)	7 ± 4.20	7.23 ± 4.29	6.85 ± 4.14	0.469
BMI (kg/m^2^)	30.08 ± 4.42	30.02 ± 4.37	30.12 ± 4.50	0.858
Diabetes duration	13.95 ± 7.08	13.44 ± 7.91	14.27 ± 6.49	0.346
≥ 1 chronic complications	132 (48.4)	46 (43.0)	86 (51.8)	0.173
Insulin users	159 (58.2)	46 (43.0)	113 (68.1)	**<0.001**
Antidepressant users	31 (11.4)	12 (11.2)	19 (11.4)	1.000
HbA1c	8.68 ± 1.71			
HADS depression	4.45 ± 3.59	3.93 ± 3.11	4.7 ± 3.84	**0.047**
HADS anxiety	7.24 ± 3.84	7.31 ± 3.94	7.19 ± 3.78	0.803
23QVS	42.70 ± 10.59	42.61 ± 9.20	42.76 ± 11.43	0.910

Data are means ± SD or n (%)*. P* values based on Student’s test or *X² test* comparing between those with HbA1c < 8 or ≥8.

Legend: *HADS:* The Hospital Anxiety and Depression Scale; 23QVS: The 23 Questions to assess Vulnerability to Stress, *BMI*: Body Mass Index; *HbA1c*: Hemoglobin A1c.

#### Predictors of poor glycemic control

After ensuring for lack of multicollinearity among variables by checking bivariate correlations (all ≤ r = 0.543, Pearson’s correlation coefficient between HADS anxiety and 23QVS), hierarchical logistic regressions were conducted (Table 2). Both significant demographic and clinical predictors for worse metabolic control (HbA1c ≥ 8) were younger and insulin users. HADS depression was associated with an increase risk of poor metabolic control in all models. Both HADS anxiety and 23QVS were not predictive of worse metabolic control.

**Table 2:** Multivariate hierarchical logistic regression with metabolic control (HbA1c <8 and ≥8) as dependent variable

	**Model 1**	**Model 2**	**Model 3**	**Model 4**
Age (years)	0.92 (0.87-0.96)**	0.91 (0.86-0.96)**	0.90 (0.85-0.95)***	0.90 (0.85-0.95)***
Gender	0.98 (0.59-1.63)	1.04 (0.61-1.78)	1.06 (0.60-1.88)	1.06 (0.60-1.88)
Education level (years)	0.96 (0.91-1.02)	0.96 (0.91-1.02)	0.97 (0.91-1.03)	0.96 (0.89-1.02)
BMI (kg/m^2^)		1.01 (0.95-1.07)	1.01 (0.95-1.07)	1.01 (0.95-1.07)
Diabetes duration		1.02 (0.98-1.06)	1.02 (0.98-1.06)	1.02 (0.98-1.06)
≥ 1 chronic complications		1.13 (0.66-1.94)	1.09 (0.63-1.90)	1.11 (0.64-1.93)
Insulin users		2.69 (1.57-4.59)***	2.64 (1.53-4.54)***	2.65 (1.54-1.58)***
Antidepressant users		1.15 (0.50-2.62)	1.06 (0.45-2.48)	1.09 (0.46-2.57)
HADS depression			1.13 (1.02-1.24)*	1.14 (1.03-1.27)**
HADS anxiety			0.92 (0.84-1.00)	0.94 (0.85-1.03)
23QVS				0.98 (0.95-1.01)
Nagelkerke R^2^	0.065	0.148	0.179	0.184
Cox&Snell R^2^	0.048	0.109	0.132	0.136
Model *X*^2^	13.4**	31.6***	38.7***	39.9***

*1*^*st*^*block:* Demographic variables: age, gender (male = 1), education; 2^nd^*block:* Demographic and Clinical variables: BMI, diabetes duration, ≥ 1 chronic complications (yes = 1), insulin users (yes = 1), antidepressants users (yes = 1); 3^rd^ block: Demographic, Clinical and Psychometric variables HADS anxiety, HADS depression; 4^th^ block: Demographic, Clinical, Psychometric variables HADS anxiety, HADS depression and 23QVS. Results: OR (95% CI).

Legend: *HADS:* The Hospital Anxiety and Depression Scale; 23QVS: The 23 Questions to assess Vulnerability to Stress, *BMI*: Body Mass Index; *HbA1c*: Hemoglobin A1c. Significance level: *P < 0.05;**P < 0.01;***P < 0.001.

### Discussion and conclusion

The aim of our cross-sectional study was to evaluate whether vulnerability to generic stress is predictive of poor metabolic control in patients with T2DM beyond depression or anxiety. Analysis was done adjusting for several demographic and clinical features, such as chronic complications, insulin use and antidepressants. In our study, vulnerability to stress was not predictive of poor metabolic control. A significant association with increased risk of worse metabolic control was shown among patients with depressive symptoms. Anxiety symptoms did not achieve a predictive value for worse glycemic control.

Our findings are consistent with other studies reporting relations between depressive symptoms and poor metabolic control in T2DM [15,16,18]. A literature meta-analysis shows a small to moderate effect-size of 0.17 [95% CI 0.13-0.21] similar in studies of type 1 or type 2 diabetes [15]. Depressive symptoms have an impact on metabolic control by behavioral or biological involvement. In a primary care sample of T2DM patients, depressive symptoms were incrementally related to poorer self-care behaviors, including lower adherence to diet, exercise recommendation and prescribed medication [35]. A biological proposed mechanism underlying depression and diabetes concerns pathophysiologic conditions such as activation of hypothalamic pituitary adrenocortical axis and sympathomedullary axis with an increased release of cortisol and catecholamines. Moreover, reduced activity associated to depression might lead to development of obesity, cytokines release and insulin resistance, even more deteriorating glycemic control [36]. Depression among T2DM patients has been described as a long lasting condition, with 58% recurrences during the first year after index depressive episode. About 79% of patients who were already depressed when recruitment took place reported at least one recurrence of depressive episode during the 5 years period the study lasted [37].

Outside distress emotional situations, vulnerability to stress may encompass two quiet different life paths. Those who are pessimistic and anxious will fail to adhere to treatment regimens, engage in unhealthy lifestyle, and have disrupted homeostasis, lack of social support and increased poor health and those who leads to neurotic vigilance, treatment adherence and better health [38]. Patients with T2DM having no psychiatric conditions and neuroticism personality traits, presented better metabolic control. Probably, the sense of distress could motivate to a better insight level regarding the need to find effective coping strategies [13]. The hypothesis of off-setting heterogeneous behaviors concerning diabetes and its treatment among vulnerable to stress patients may explain the lack of association with metabolic control found in our data. Emotion-focused coping alone, usually more associated to less emotionally stable subjects, is associated to negative health outcomes. However, when emotion-focused coping precedes and fosters problem-focused coping which begins afterwards, it leads to increased medical adherence [39]. Otherwise in a study designed to predict which T2DM patients would have better response to addition of relaxation training in their standard intensive therapy, none of the individual differences related to stress reactivity explained the variability in the changes in HbA1c [8]. In another study, a significant reduction of 0.5% in HbA1c was achieved after 12-month follow-up in the group receiving stress management treatment against the group receiving diabetes education alone, but stress-responsiveness individual features were not associated with this result [7]. This suggests that some mediator factor between vulnerability to stress and glycemic control was not evaluated or reflects a dynamic changing interaction with contingent strategies between an individual and the circumstances [40]. Type 2 Diabetes as an “if-then” disease in daily life adaptation may fit well with this last statement that may be rather difficult to capture in research. We argue that when people with vulnerability to stress get depressed, adaptation to generic or specific stressors becomes more ineffective and enduring with negative consequences to glycemic outcome by behavioral or biologic pathways.

No association of anxiety symptoms to worse metabolic control was found. Anxiety has been associated to metabolic control, although with conflicting results. In a prospective study with T2DM patients, anxiety seemed to facilitate earlier detection of diabetes, however 6 months afterwards impact of diagnosis was stronger in anxious individuals [41]. In a meta-analysis with type 1 or 2 diabetes patients, only anxiety disorders were associated to hyperglycemia, but not sub-clinical anxiety symptoms [17]. Nevertheless, in recent studies, T2DM patients did not present an association regarding their anxiety disorder or symptoms with HbA1c [18,21].

In clinical practice, our results come up with the relevance of assessing depression in T2DM patients to better approach glycemic control deterioration. Patients with vulnerability to stress were not associated with increased risk of worse metabolic control, but they represent a population with a greater tendency to become depressed. A closer monitoring with regular depression assessment is advisable with such vulnerable patients.

Several limitations should be addressed to our study. The cross-sectional nature of the study prohibits any statements with respect to causality. Being in poor glycemic control over longer periods of time may also cause serious distress and depression [27,33]. Prospective studies are desirable to clarify the particular role and implication of vulnerability to generic stress to metabolic control in symptomatic or asymptomatic patients and clarify what are the possible mediators involved. We used continuous scores concerning depression, but a diagnostic psychiatric interview remains the gold standard in research and clinical practice.

The most robust finding of our study was that depressive symptoms are associated with glycemic control but vulnerability to stress is not.

Further studies are needed to better clarify the relationship between anxiety symptoms and metabolic control in these patients.

## Abbreviations

APDP, Portuguese Diabetes Association; T2DM, Type 2 Diabetes Mellitus; HADS, The Hospital Anxiety Depression Scale; 23QVS, The 23 Questions to assess Vulnerability to Stress; BMI, Body Mass Index; HbA1c, Hemoglobin A1c; SPSS, Statistical Package for Social Sciences.

## Competing interests

VVD: *Employment:* Consultant for Angelini Pharmaceuticals. *Research grant:* Roche diagnostics. *Other research grant or medical continuous education:* Sanofi-Aventis, AstraZeneca, Bristol-Myers-Squibb, Janssen, and Lundbeck.

The authors CG, JFR, IC and AB declare that they have no competing interests.

## Authors’ contributions

CG, VVD, JFR, IC, AB were responsible for the initial design of the study. CG did the analyses and wrote the first draft of the paper. VVD contribute to the analysis, interpretation of the results and the review of the drafts. All authors contributed to the interpretation of the data and review of the manuscript for important intellectual content. All authors read and approved the final manuscript.
